# Fitting Gaussian mixture models on incomplete data

**DOI:** 10.1186/s12859-022-04740-9

**Published:** 2022-06-01

**Authors:** Zachary R. McCaw, Hugues Aschard, Hanna Julienne

**Affiliations:** 1 School of Public Health, Harvard T.H. Chan, 677 Huntington Ave, Boston, MA 02115 USA; 2 Department of Computational Biology, Institut Pasteur, Université de Paris, 25-28 Rue du Dr Roux, 75015 Paris, France

**Keywords:** Clustering, Missing data, Gaussian mixture models

## Abstract

**Background:**

Bioinformatics investigators often gain insights by combining information across multiple and disparate data sets. Merging data from multiple sources frequently results in data sets that are incomplete or contain missing values. Although missing data are ubiquitous, existing implementations of Gaussian mixture models (GMMs) either cannot accommodate missing data, or do so by imposing simplifying assumptions that limit the applicability of the model. In the presence of missing data, a standard *ad hoc* practice is to perform complete case analysis or imputation prior to model fitting. Both approaches have serious drawbacks, potentially resulting in biased and unstable parameter estimates.

**Results:**

Here we present missingness-aware Gaussian mixture models (MGMM), an R package for fitting GMMs in the presence of missing data. Unlike existing GMM implementations that can accommodate missing data, MGMM places no restrictions on the form of the covariance matrix. Using three case studies on real and simulated *’omics* data sets, we demonstrate that, when the underlying data distribution is near-to a GMM, MGMM is more effective at recovering the true cluster assignments than either the existing GMM implementations that accommodate missing data, or fitting a standard GMM after state of the art imputation. Moreover, MGMM provides an accurate assessment of cluster assignment uncertainty, even when the generative distribution is not a GMM.

**Conclusion:**

Compared to state-of-the-art competitors, MGMM demonstrates a better ability to recover the true cluster assignments for a wide variety of data sets and a large range of missingness rates. MGMM provides the bioinformatics community with a powerful, easy-to-use, and statistically sound tool for performing clustering and density estimation in the presence of missing data. MGMM is publicly available as an R package on CRAN: https://CRAN.R-project.org/package=MGMM.

**Supplementary Information:**

The online version contains supplementary material available at 10.1186/s12859-022-04740-9.

## Background

Gaussian mixture models (GMMs) provide a flexible approach to multivariate density estimation and probabilistic clustering [1]. Most implementations of GMMs in the R programming language, including mclust [2] and mixtools [3], require complete data. The few implementations that do allow for missing values, such as MixAll [4], have limited applicability due to their restrictive simplifying assumptions. For example, MixAll assumes diagonal covariance matrices, which implies that the elements of the Gaussian vectors under consideration are mutually independent. In practice, both correlated and missing data are common. Our work was motivated by the problem of clustering summary statistics arising from genome-wide association studies (GWAS) of multiple correlated traits [5]. Missing data arose because not every genetic variant was tested for association with every trait.

Although commonly applied, standard approaches for addressing missing data prior to clustering, including complete case analysis and imputation, have serious drawbacks. By discarding information from observations that are only partially observed, complete case analysis makes inefficient use of the data. This leads to unstable estimates of model parameters and cluster assignments that are susceptible to significant changes if the missingness pattern of the input data changes slightly. On the other hand, mean or median imputation introduces bias by making the incomplete observations appear less variable, and by shrinking the incomplete observations towards the complete data. This can result in inaccurate posterior membership probabilities that place excess weight on clusters with less missing data. Although a method has been described for estimating GMMs from incomplete data [6], there are no existing implementations in R.

To fill this gap, we present MGMM [7], a computationally efficient R package for maximum likelihood estimation of GMMs in the presence of missing data. Our package is carefully implemented and documented for ease of use. In contrast to complete case analysis, our approach makes full use of the available data; and in contrast to clustering after imputation, our approach is unbiased for estimating the parameters of the generative GMM, accurately assesses the posterior membership probabilities, and correctly propagates estimation uncertainty. Moreover, our implementation places no restrictions on the model’s covariance structures.

MGMM employs an expectation conditional maximization (ECM) algorithm [8], which accelerates estimation by breaking direct maximization of the EM objective function into a sequence of simpler conditional maximizations, each of which is available in closed form. While EM algorithms are regularly used for estimating GMMs, for example by both mclust and mixtools, those implementations only address missingness of the true cluster assignments, and not missingness of elements from the input vectors. In contrast, our ECM algorithm handles both missingness of the cluster assignments and of elements from the input data. We present a comprehensive benchmark, including three case studies, demonstrating that when the underlying distribution is well-approximated by a GMM, MGMM is better able to recover the true cluster assignments than MixAll or than standard GMM applied after state of the art imputation (e.g. multiple imputation by chained equations, MICE [9]). While we prioritized cluster assignments accuracy, our implementation also proves competitive in regard to running time for the missingness rates usually encountered in real data.

## Methods

### Model

This section provides an overview of the statistical model. For a detailed derivation and description of the ECM algorithm, see the Supporting Information.

#### Statistical model overview

Consider *n* independent vectors ${\varvec{y}}_{i}=\text {vec}(Y_{i1},\ldots ,Y_{id})$ in ${\mathbb {R}}^{d}$, each arising from one of *k* distinct clusters. Although *k* is assumed known throughout this work, see "Methods" section of the Supporting Information for an approach to choosing *k*. Let $Z_{ij}=1$ if the *i*th observation belongs to cluster *j*, and define the $k\times 1$ indicator vector ${\varvec{z}}_{i}=\text {vec}(Z_{i1},\ldots ,Z_{ik})$. Conditional on membership to the *j*th cluster, ${\varvec{y}}_{i}$ follows a multivariate normal distribution, with cluster-specific mean ${\varvec{\mu }}_{j}$ and covariance ${\varvec{\Sigma }}_{j}$. Let $\pi _{j}$ denote the marginal probability of membership to the *j*th cluster. The observations can be viewed as arising from the following hierarchical model:

1 $$\begin{aligned} \begin{aligned} {\varvec{z}}_{i} \sim \text {Multinomial}(1,{\varvec{\pi }}), \\ {\varvec{y}}_{i}\big |(Z_{ij}=1) \sim N\big ({\varvec{\mu }}_{j},{\varvec{\Sigma }}_{j}\big ). \end{aligned} \end{aligned}$$

Marginalized over the latent cluster assignment vector ${\varvec{z}}_{i}$, each observation ${\varvec{y}}_{i}$ follows a *k* component Gaussian mixture model (GMM):

2 $$\begin{aligned} f({\varvec{y}}_{i}) = \sum _{j=1}^{k}\pi _{j}f\big ({\varvec{y}}_{i}|{\varvec{\mu }}_{j},{\varvec{\Sigma }}_{j}\big ). \end{aligned}$$

To perform estimation in the presence of missingness, we derive the EM objective function $Q({\varvec{\pi }},{\varvec{\theta }}|{\varvec{\pi }}^{(r)},{\varvec{\theta }}^{(r)})$, which is the expectation of the complete data log likelihood, given the observed data and current parameter estimates (see the Supporting Information for complete derivation). The EM objective is optimized using a sequence of three conditional maximizations. Let ${\hat{\gamma }}_{ij}^{(r)}$ denote the *responsibility* of the *j*th cluster for the *i*th observation, which is the current conditional probability of membership to that cluster, given the observed data. In the first step, the cluster means are updated using the responsibility-weighted average of the working outcome vectors $\hat{{\varvec{y}}}_{ij}^{(r)}$. In the next step, the cluster covariances are updated using the responsibility-weighted average of the working residual outer products. In the final step, the cluster responsibilities and marginal membership probabilities are updated using the new means and covariances. This process iterates until the improvement in the EM objective drops below the specified tolerance. Unbiased estimation of the model parameters requires that missingnesss in the outcome vector occur at random [10]. This means that whether a particular element of the outcome vector is missing can depend on the values of those elements that are observed, but not upon the values of those elements that are missing. See section 1.3 of the Supporting Information for further discussion of the missing at random assumption.

#### Imputation

Having fit the GMM in (2) via maximum likelihood, the missing values ${\varvec{y}}_{i}^{\text {miss}}$ of each observation ${\varvec{y}}_{i}$ may subsequently be imputed. Note that, in contrast to the imputation *before* estimation procedure commonly used to address missing input data, MGMM performs imputation only *after* estimation. In this way, imputation has no effect on the final maximum likelihood estimates $(\hat{{\varvec{\pi }}},\hat{{\varvec{\theta }}})$. To perform a deterministic single imputation, as is done by the FitGMM function, $\hat{{\varvec{y}}}_{i}^{\text {miss}}$ may be set to its posterior expectation given ${\varvec{y}}_{i}^{\text {obs}}$:

3 $$\begin{aligned} \hat{{\varvec{y}}}_{i}^{\text {miss}}&\equiv {\mathbb {E}}\left( {\varvec{y}}_{i}^{\text {miss}}|{\varvec{y}}_{i}^{\text {obs}};\hat{{\varvec{\pi }}}, \hat{{\varvec{\theta }}}\right) \\&= {\mathbb {E}}\left\{ {\mathbb {E}}\left( {\varvec{y}}_{i}^{\text {miss}}|z_{ij}=1,{\varvec{y}}_{i}^{\text {obs}};\hat{{\varvec{\pi }}}, \hat{{\varvec{\theta }}}\right) \right\} \\&= \sum _{j=1}^{k}\hat{{\varvec{y}}}_{ij}^{\text {miss}}{\hat{\gamma }}_{ij}. \end{aligned}$$

Here ${\hat{\gamma }}_{ij}$ is the final responsibility of cluster *j* for observation *i*, and:

$$\begin{aligned} \hat{{\varvec{y}}}_{ij}^{\text {miss}} = \hat{{\varvec{\mu }}}_{\text {miss},j} + \hat{{\varvec{\Sigma }}}_{\text {miss},\text {obs},j}\hat{{\varvec{\Sigma }}}_{\text {obs},j}^{-1}\left( {\varvec{y}}_{i}^{\text {obs}} - \hat{{\varvec{\mu }}}_{\text {obs},j}\right) . \end{aligned}$$

While single imputation to the posterior expectation is useful for visualization, drawing inferences from singly-imputed data is generally invalid [10]. For subsequent inference, a multiple imputation procedure is necessary, wherein multiple stochastic imputations of the input data are generated, analyzed in parallel, and the resulting estimates combined. To generate a single stochastic imputation of ${\varvec{y}}_{i}^{\text {miss}}$, as is done by the GenImputation function, the latent cluster membership ${\varvec{z}}_{i}$ is first drawn from a multinomial distribution over $\hat{{\varvec{\gamma }}}_{i} = {\mathbb {P}}({\varvec{z}}_{i} | {\varvec{y}}_{i}^{\text {obs}}; \hat{{\varvec{\pi }}}, \hat{{\varvec{\theta }}})$:

$$\begin{aligned} {\varvec{z}}_{i} \sim \text {Multinomial}(1, \hat{{\varvec{\gamma }}}_{i}). \end{aligned}$$

Given the cluster assignment, $Z_{ij} = 1$, the missing elements ${\varvec{y}}_{i}^{\text {miss}}$ are drawn from a normal distribution with mean:

$$\begin{aligned} {\mathbb {E}}\left( {\varvec{y}}_{i}^{\text {miss}} | {\varvec{y}}_{i}^{\text {obs}}, Z_{ij} = 1; \hat{{\varvec{\pi }}}, \hat{{\varvec{\theta }}}\right) = \hat{{\varvec{\mu }}}_{j}^{\text {miss}} + \hat{{\varvec{\Sigma }}}_{\text {miss},\text {obs},j}\hat{{\varvec{\Sigma }}}_{\text {obs},\text {obs},j}\left( {\varvec{y}}_{i}^{\text {obs}} - \hat{{\varvec{\mu }}}_{\text {obs},j}\right) \end{aligned}$$

and covariance:

$$\begin{aligned} {\mathbb {V}}\left( {\varvec{y}}_{i}^{\text {miss}} | {\varvec{y}}_{i}^{\text {obs}}, Z_{ij} = 1; \hat{{\varvec{\pi }}}, \hat{{\varvec{\theta }}}\right) = \hat{{\varvec{\Sigma }}}_{\text {miss},\text {miss},j} - \hat{{\varvec{\Sigma }}}_{\text {miss}, \text {obs}, j} \hat{{\varvec{\Sigma }}}_{\text {obs}, \text {obs}, j}^{-1}\hat{{\varvec{\Sigma }}}_{\text {obs}, \text {miss}, j} \end{aligned}$$

An exposition of how to use multiple stochastic imputations for inference is presented in the Supporting Information.

### Benchmarking method

All analyses were performed in R 3.5.0 R [11]. We designed a benchmarking procedure to compare the performance of MGMM against imputation followed by standard GMM (also implemented by MGMM) and another package that allows for missing values (MixAll). The imputation methods included in the benchmark were: naive mean and median imputation; k-nearest neighbors imputation, as implemented by the VIM package [12]; multiple imputation by chained equations, as implemented by the MICE package [9]; and random forest imputation, as implemented by the missforest package [13]. We defined clustering performance as the capacity of the algorithm to recover the true cluster assignments when applied to example data sets. We assessed the quality of the clustering by calculating the adjusted rand index (ARI) between the recovered and true class assignments. The running time was defined as the time necessary to obtain cluster assignation starting with the data set with missing values. We applied the benchmarking procedure to four case studies: a simulated four component mixture of bivariate Gaussians, a cancer patient RNA-seq data set, simulated genome-wide association studies (GWAS) summary statistics, and summary statistics from GWAS for cardiovascular disease risk factors [14].

#### Missingness

For *n* observations on *d* dimensional data, a fraction of missing values *m* was introduced completely at random by setting $\lceil (m\times n \times d)\rceil$ elements of the data set to NA.

#### Evaluation metric

The quality of clustering was evaluated using the ARI [15, 16]. Briefly, the Rand Index (RI) is a measure of similarity that assesses the agreement between two partitions of a collection of *n* objects. All possible pairs of objects are examined, and the proportion of pairs that are either 1. in the same cluster or 2. in different clusters according to both partitions is calculated. ARI is a variation of the RI that is adjusted for chance, and is permutation invariant. A value near zero suggests the agreement between the two partitions is no better than expected by chance, while a value of one occurs when the two partitions are identical. We define the quality of clustering as the ARI between the reference or true clustering, established in the data set description, and the clustering performed in the presence of missingness.

#### Benchmarking procedure

**Fig. 1:**
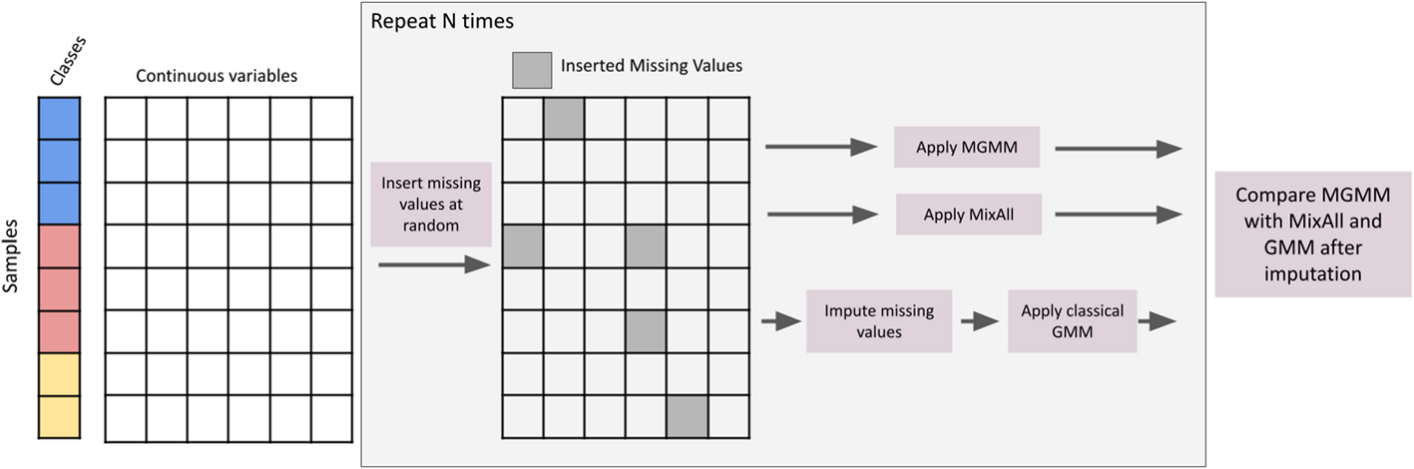
Benchmark procedure schematic. The input data are continuous vectors with known class assignments. Missing values are introduced completely at random. GMMs were then fit to the incomplete data in several ways: 1. by using MGMM, which allows for missing values and arbitrary covariance structures; 2. by using MixAll, which allows for missing values but assumes a diagonal covariance structure; 3. by imputing the missing values, then fitting a standard GMM. The GMMs were evaluated based on the adjusted Rand index between the predicted and true cluster assignments. This procedure was repeated $N=$ times

We designed the benchmarking procedure outlined in Fig. 1 and described in Algorithm 1 to compare the performance of MGMM with imputation followed by standard GMM.

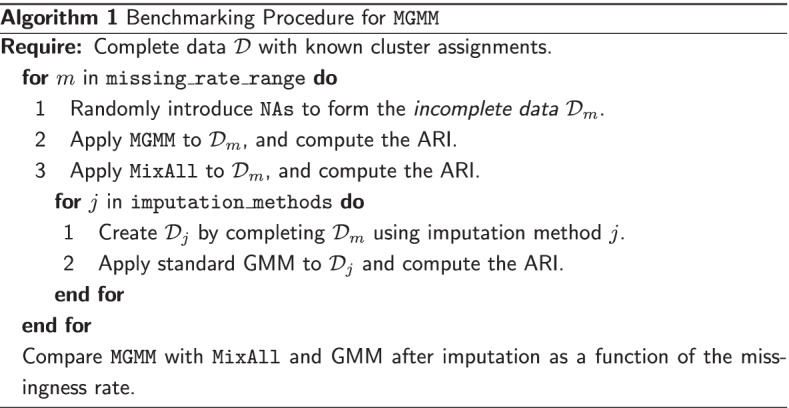



### Benchmark data sets

#### Simulated Gaussian mixture

For the first clustering task, we consider data that were truly generated from a GMM, which is the setting in which MGMM should perform optimally. Data were simulated according to the hierarchical model in Eq. (1). The dimensionality *d* was set to 2 and the number of cluster components *k* to 4. The marginal density of the data generating process was:

$$\begin{aligned} \left( {\begin{array}{c}Y_{i1}\\ Y_{i2}\end{array}}\right) \sim \sum _{j=1}^{4}\pi _{j}N({\varvec{\mu }}_{j},{\varvec{\Sigma }}_{j}). \end{aligned}$$

The means (${\varvec{\mu }}_{j}$) were drawn from a uniform distribution on the square:

$$\begin{aligned} \{(x,y): -5\le x\le 5, -5\le y \le 5\}. \end{aligned}$$

The component variances were set to 0.9:

$$\begin{aligned} \Sigma _{11,j} = {\mathbb {V}}(Y_{i1}|Z_{ij}=1) = \Sigma _{22,j} = {\mathbb {V}}(Y_{i2}|Z_{ij}=1) = 0.9. \end{aligned}$$

The covariance was uniformly sampled from the interval $(-0.9,0.9)$:

$$\begin{aligned} \Sigma _{12,j} = {\mathbb {C}}(Y_{i1},Y_{i2}|Z_{ij}=1) \sim U(-0.9, 0.9). \end{aligned}$$

Marginal membership to each cluster was equally likely, $\pi _{j}=0.25$ for $j\in \{1,\ldots ,4\}$. A sample of size $n=2000$ was generated using the rGMM function from MGMM. The true (generative) component memberships $({\varvec{z}}_{i})$ were used as the reference when evaluating clustering performance on incomplete data.

#### RNA sequence data from cancer patients

For the second clustering task, cancer gene expression data [17] were retrieved from the University of California Irvine machine learning repository. These data consist of expression values for 20,531 genes from $n=801$ patients having 1 of $k=5$ tumor types. Marginal analysis of variance was performed to identify the 20 most significantly differentially expressed genes (DEGs) across tumor types. The patient by DEG matrix was then decomposed via principal components analysis (PCA), and the final cluster task was performed on the $d=5$ leading principal components. $Y_{il}$ represents the expression of patient *i* along the *l*th principal component. The patient’s observed tumor type was used as the reference when evaluating clustering performance on incomplete data. The tumor types are abbreviated as follows:

- BRCA: Breast carcinoma.
- COAD: Colon adenocarcinoma.
- KIRC: Kidney renal clear-cell carcinoma.
- PRAD: Prostate adenocarcinoma.
- LUAD: Lung adenocarcinoma.

#### GWAS summary statistics

For the third clustering task, we consider summary statistics, both simulated and real, arising from GWAS for cardiovascular disease risk factors. In this setting, *i* indexes single nucleotide polymorphisms (SNPs) and $Y_{il}$ is the standardized score (i.e. Z-score) quantifying the magnitude of the observed association between SNP *i* and phenotype *l*. The SNPs may belong to one of *k* clusters, where the Z-scores of SNPs within a cluster may exhibit correlations due to the combination of environmentally-induced correlation of the traits and sample overlap between the GWAS in which the Z-scores were ascertained.

A simulated set of GWAS summary statistics was generated for $d=3$ traits and 900 SNPs arising from 1 of $k=3$ clusters. The marginal density was:

$$\begin{aligned} \begin{pmatrix} Y_{i1} \\ Y_{i2} \\ Y_{i3} \end{pmatrix} \sim \sum _{j=1}^{3}\pi _{j}N({\varvec{\mu }}_{j},{\varvec{\Sigma }}_{j}). \end{aligned}$$

The mean vectors (${\varvec{\mu }}_{j}$) were set to zero, and the cluster covariances (${\varvec{\Sigma }}_{j}$) were set to:

$$\begin{aligned} {\varvec{\Sigma }}_{1}&= \begin{pmatrix} 2.5 & 2 & 0\\ 2 & 2.5 & 0 \\ 0 & 0 & 0.3 \end{pmatrix}\\ {\varvec{\Sigma }}_{2}&= \begin{pmatrix} 2.25 & -2 & 0\\ -2 & 2.25 & 0 \\ 0 & 0 & 0.3 \end{pmatrix}\\ {\varvec{\Sigma }}_{3}&= \begin{pmatrix} 0.2 & 0 & 0\\ 0 & 0.2 & 0 \\ 0 & 0 & 4.5 \end{pmatrix}. \end{aligned}$$

**Fig. 2:**
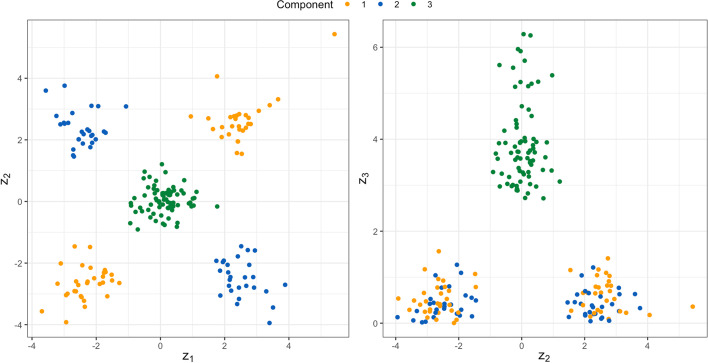
Scatter Plot of the Simulated GWAS-like Multivariate Z-scores. Observations are colored according to component membership. The left panel plots the second coordinate against the first, and the right panel plots the third coordinate against the second

Marginal membership to each cluster was equally likely $\pi _{j}=0.33$ for $j\in \{1,\ldots ,3\}$. These covariance structures were chosen to represent a variety of situations: (1) pleiotropic SNPs whose effects are positively correlated for the two first traits (${\varvec{\Sigma }}_{1}$); (2) pleiotropic SNPs whose effects are negatively correlated for the two first traits SNPs (${\varvec{\Sigma }}_{2}$); (3) SNPs acting predominantly on the third traits (${\varvec{\Sigma }}_{3}$). A sample of size $n=900$ was generated using the rGMM function from the MGMM package. To emulate the omission of non-significant results, which frequently occurs when reporting GWAS summary statistics, the SNPs were filtered to those having evidence of association with the traits at $p \le 0.05$ via the omnibus test (9) detailed in the appendix. After filtering, $n=183$ SNPs remained, with marginal cluster frequencies: $\pi _1 = 0.25$, $\pi _2 = 0.42$, $\pi _3 = 0.33$. The topology of the resulting data set is presented in Fig. 2. The true (generative) component memberships $({\varvec{z}}_{i})$ were used as the reference when evaluating clustering performance on incomplete data.

A set of real GWAS summary statistics for cardiovascular disease risk factors was prepared as described in [14]. These traits were: body mass index (BMI), coronary artery disease (CAD), low density lipoprotein (LDL), triglycerides (TG), waist to hip ratio (WHR), and any strokes (AS). From this collection of traits, we formed three example data sets. The first included $\{$BMI, CAD, LDL$\}$ only; the second included $\{$LDL, TG, BMI, AS, CAD$\}$; the third $\{$LDL, TG, BMI, AS, WHR$\}$. We selected independent SNPs that were genome-wide significant (p-value $\le 10^{-8}$) either marginally or via the omnibus test (9). These data sets contained 165, 166 and 179 SNPs respectively. For each example, a GMM with $k=3$ components was fit to the complete data (using FitGMM from the MGMM package), and the cluster assignments from this initial model were used as the reference when evaluating clustering performance on incomplete data. Because the reference clustering partition was directly derived from the data, the benchmark on these examples assess the robustness of the clustering rather than the ability to recover a true, underlying data class assignment.

### Imputation methods and **MixAll** parameter settings

Naive mean or median imputation refers to simply setting a missing value to the mean or median of the observed values along that coordinate. For kNN, a missing value was imputed to the (Euclidean) distance-weighted average of the 5 nearest observations with observed data along that coordinate. For MICE, a missing value was imputed to its conditional expectation given the observed coordinates via the method of predictive mean matching; the number of imputations was 10, and the maximum number of Gibbs sampling iterations was 50. For random forest imputation, the number of trees per forest was 100, and the maximum number of refinement iterations was 10. For MixAll, the default parameters of the clusterDiagGaussian function were adopted; this resulted in all available models being fit, and the best model, according to the integrated completed likelihood criterion, being returned.

### Filtering unassignable observations from MGMM and MICE

Since both MGMM and MICE provide an indication of the uncertainty in the cluster assignments, we created an additional clustering method in which observations with high assignment uncertainty were regarded as unassignable. This occurs when an observation could very plausibly has originated from more than one of the clusters, and may be exacerbated by excess missing data along a coordinate that helps to differentiate among clusters. This uncertainty can be assessed via the entropy of the posterior membership probabilities:

4 $$\begin{aligned} H({\varvec{y}}_{i}) = \frac{1}{\ln (k)}\sum _{j=1}^{k}{\hat{\gamma }}_{ij}(-1)\ln ({\hat{\gamma }}_{ij}), \end{aligned}$$

where ${\hat{\gamma }}_{ij}$ is the final responsibility of cluster *j* for observation *i*.

For MGMM, the entropy of the posterior cluster responsibilities is calculated by FitGMM using (4). For MICE, each input data set is multiply imputed, and each of these imputed data set results in one *maximum a posteriori* cluster assignment. The posterior probability of membership to each cluster (i.e. the responsibilities, ${\hat{\gamma }}_{ij}$) may be approximated by the proportion of imputations on which an observation was assigned to each cluster.

In the filtered versions of MGMM and MICE, observations with high assignment uncertainty are identified via entropy and removed from consideration. For a given data set, such as the Cancer RNA-Seq data set, the distribution of entropy for MICE was typically right skewed (Supplementary Fig. 3-A). Consequently, for a fixed entropy threshold, the fraction of observations deemed unassignable is systematically higher for MICE than for MGMM (see Supplementary Fig. 3-B). To conduct a fair comparison of the two methods, we proceeded as follows:

1. For MICE, filter out observations with entropy exceeding 0.2 and assess performance on the remaining data.
2. Find the proportion of observations discarded by MICE.
3. Set an entropy threshold for MGMM such that the same proportion of observations is excluded as was removed for MICE.
4. Filter out observations with entropy exceeding the MGMM threshold and assess performance on the remaining data.

This procedure provides a fair comparison of MGMM-filtered and MICE-filtered by adaptively selecting the entropy threshold for MGMM in such a way that both methods remove the same fraction observations with high assignment uncertainty.

## Benchmark results

### Four component mixture of bivariate Gaussians

**Fig. 3:**
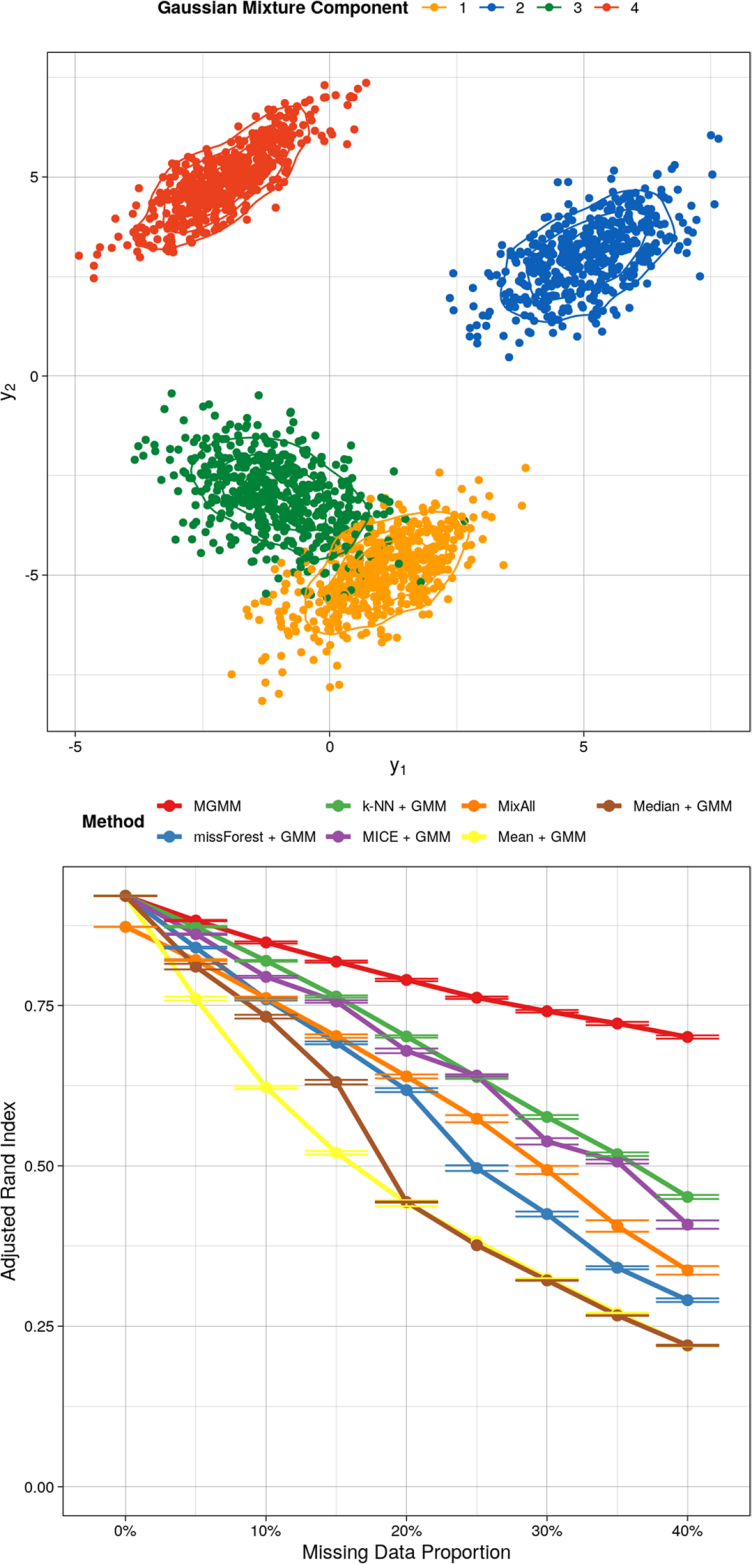
Benchmarking for the Mixture of Gaussians Data Set. The top panel includes the observations as simulated, colored according to the mixture component. The bottom panel presents the adjusted Rand index as a function of the missing data proportion for 8 different approaches to handling missing data; a higher value indicates better agreement between the predicted and true cluster assignments, adjusting for chance. Error bars represent the standard error of the mean across 20 simulation replicates

When the underlying distribution was in fact a GMM, MGMM uniformly dominated imputation plus GMM (Fig. 3) at recovering the true cluster assignments. GMM after imputation by kNN and GMM after MICE performed similarly. The performance of MixAll was relatively poor compared to MGMM, despite the data having truly been generated from a GMM. This underscores the disadvantages of an estimation procedure that incorrectly assumes a diagonal covariance structure. Interestingly, although non-parametric, random forest imputation was not competitive when the true data generating process was a GMM. Naive mean and median imputation strongly under-performed, and at elevated missingness created singularities in the data set that prevented the GMM from converging.

### RNA sequence data from cancer patients

**Fig. 4:**
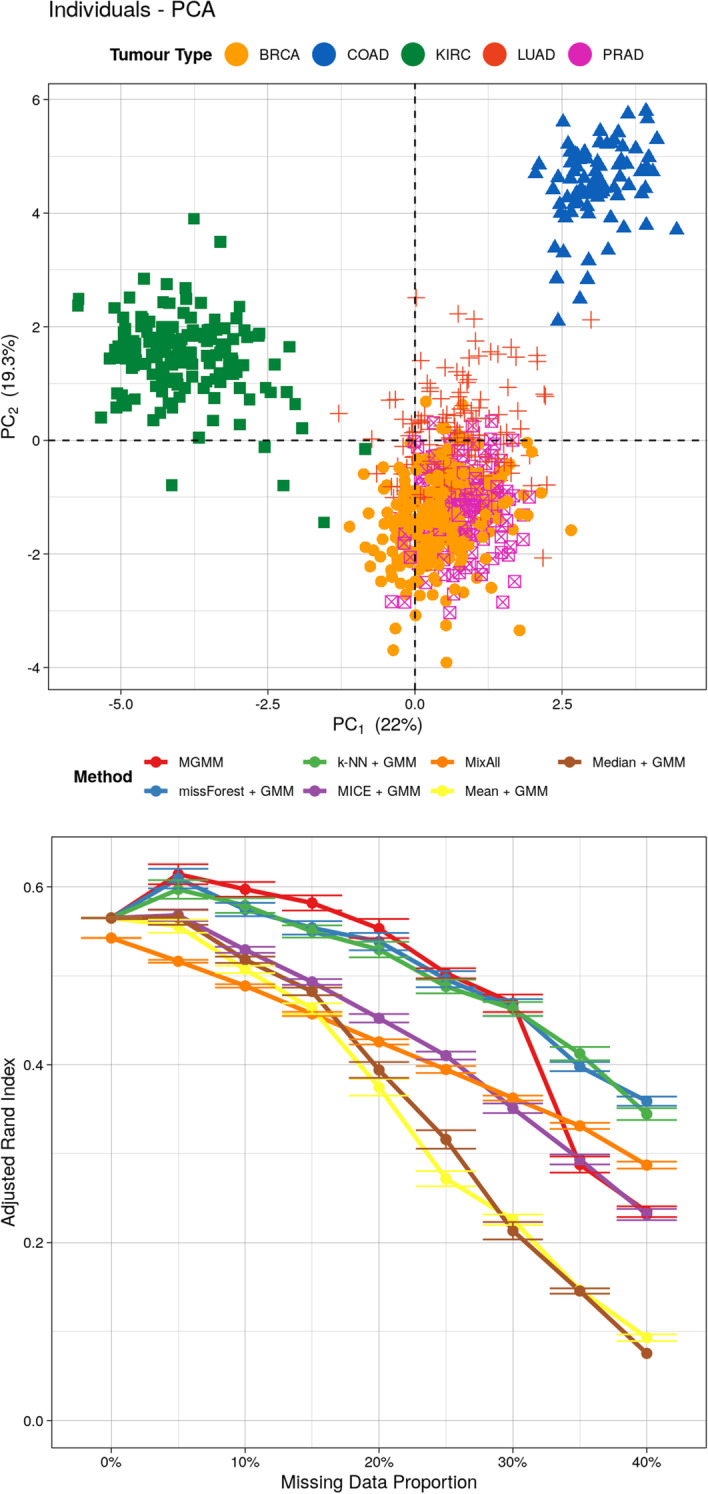
Benchmarking for the Cancer RNA-Seq Data Set. The top panel includes the projection of the expression data for $n=801$ cancer patients onto the first two principal components. Observations are colored according to tumor type. The bottom panel presents the adjusted Rand index as a function of the missing data proportion; a higher value indicates better agreement between the predicted and true cluster assignments, adjusting for chance. Error bars represent the standard error of the mean across 20 simulation replicates

For the Cancer RNA-Seq data set, where the true generative model is unlikely to be a GMM, MGMM remained highly effective at recovering the true tumor type of the patient (see Fig. 4). MixAll evinced the worst performance when the proportion of missing data was $\le 15\%$, however its performance deteriorated more slowly than the other methods, allowing it to become competitive when the proportion of missing data surpassed 35%. This may be because the simpler model assumed by MixAll is easier to fit when the data set is small and the proportion of missing values high. Random forests and kNN + GMM were competitive with MGMM, and outperformed when the proportion of missing data was $\ge 35\%$. Mean and median imputation were again not competitive, particularly when the proportion of missing data was $\ge 20\%$. MICE performed only slightly better than mean and median imputations. Linear imputation method may be ill-suited for separating the BRCA, LUDA, and PRAD tumor types.

### GWAS summary statistics

Finally, we considered clustering vectors of GWAS summary statistics arising when the same SNPs are tested for association with multiple traits. This analysis is of interest for identifying pleiotropy, individual SNPs that have effects on multiple traits, and polygenicity, collections of multiple SNPs that have effects on common traits. Such analyses are often performed by combining data from multiple independent studies, and missingness arises because not all SNPs or all traits were ascertained in all studies. Further, this analysis would generally only include SNPs that were significantly associated with at least one trait.

**Fig. 5:**
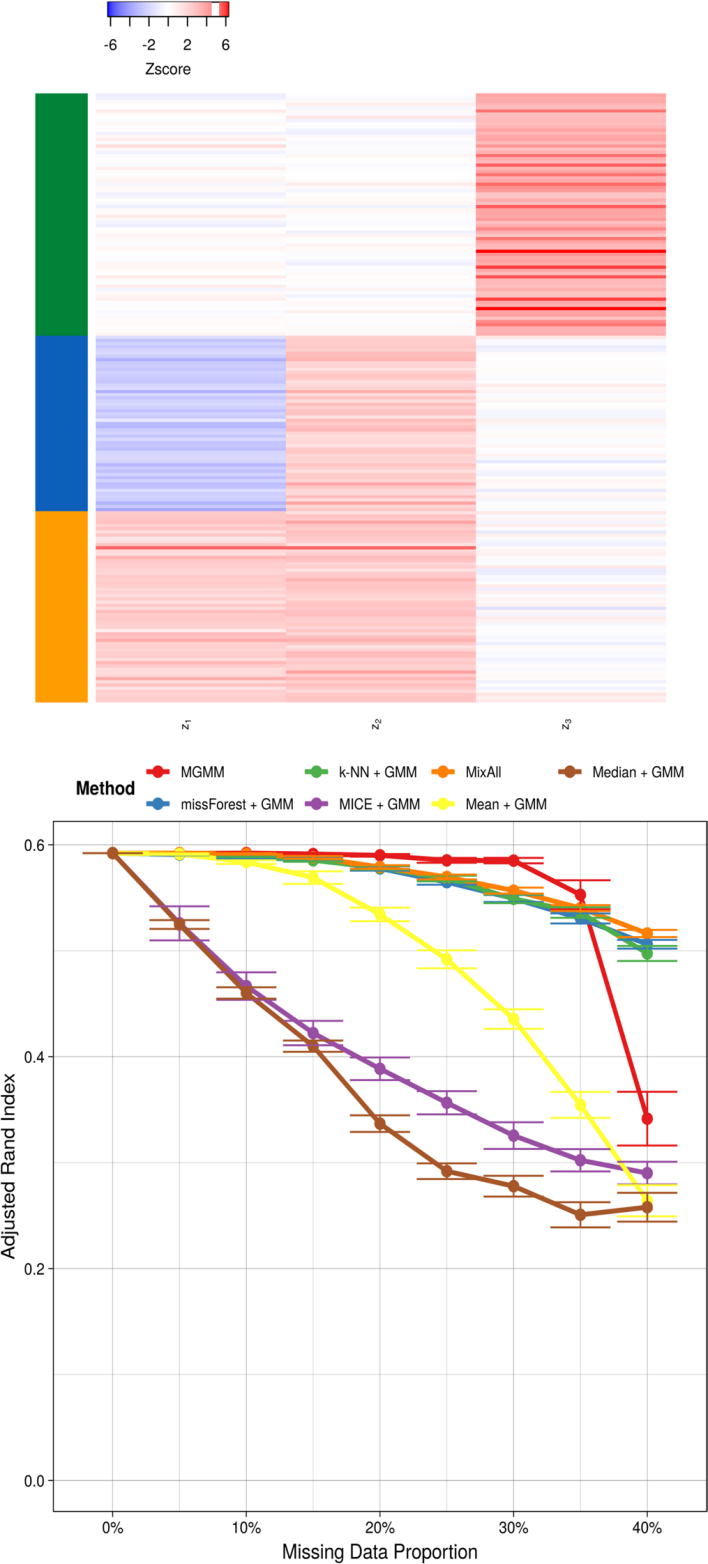
Benchmarking Simulated Multi-trait GWAS Summary Statistics. The left panel presents a heat map colored according to the normalized genetic effect, with SNPs as rows and traits as columns. The colorbar on the left represents the true cluster assignments. The right panel presents the adjusted Rand index as a function of the missing data proportion; a higher value indicates better agreement between the predicted and true cluster assignments, adjusting for chance. Error bars represent the standard error of the mean across 20 simulation replicates

Here we discuss one simulated and one real data example; two additional real data examples are presented in the appendix. For the simulated summary statistics in Fig. 5, the clustering task is same as the one presented on Fig. 3. The three clusters are clearly separated. Yet, the task remains challenging due to the specific and unusual cluster topology arising from GWAS data. Since the underlying distribution was in fact a GMM, MGMM again performs very well, only falling off when the proportion of missing data reaches $\ge 40\%$. In this example, MixAll performed was competitive with MGMM, although MGMM did outperform until the proportion of missing data become high. For this simulation, the data generation process MixAll because the covariance structure is truly diagonal for the 3rd component of the mixture, and close to diagonal for the 2nd. As for the RNA-Seq example (Fig. 4), random forest and kNN were competitive with MGMM, outperforming at very high missingness. Surprisingly, MICE under-performed naive mean imputation, and was comparable to native median imputation.

**Fig. 6:**
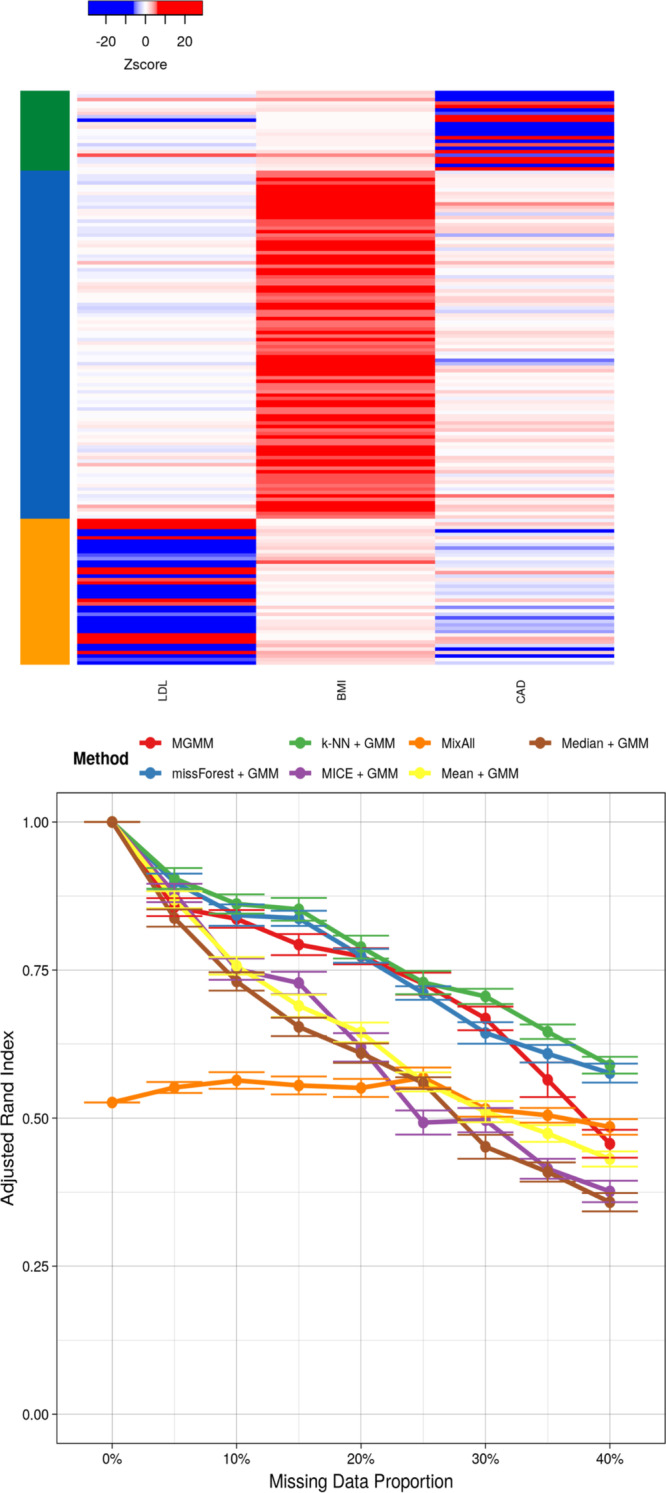
Benchmarking Real Multi-trait GWAS Summary Statistics for 3 Cardiovascular Risk Factors. These were body mass index (BMI), coronary artery disease (CAD), and low density lipoprotein (LDL). The left panel presents a heat map colored according to the standardized genetic effect, with SNPs as rows and traits as columns. The colorbar on the left represents the true cluster assignments. The right panel presents the adjusted Rand index as a function of the missing data proportion; a higher value indicates better agreement between the predicted and true cluster assignments, adjusting for chance. Error bars represent the standard error of the mean across 20 simulation replicates

An analogous clustering task applied to summary statistics from real GWAS of BMI, CAD, and LDL is presented in Fig. 6. The three clusters, identified by applying a 3-component GMM to the data before the introduction of missingness, appear well-differentiated on the heat map. kNN and random forests offered the best performance, followed by MGMM, whose performance deteriorated at missingness $\ge 35\%$. The deficit in performance of MGMM compared to kNN and random forests, even at low missingness, likely reflects a departure of the true data generating process from a GMM. Similarly, this departure likely explains the overall lower performance of MixAll for these data. As in the case of simulated GWAS summary statistics, MICE was not competitive, performing similarly to naive mean and median imputation. Two alternative examples with different set of traits are presented in Supplementary Material (see Supplementary Figs. 1 and 2).

### Comparison of MICE-filtered and MGMM-filtered

**Fig. 7:**
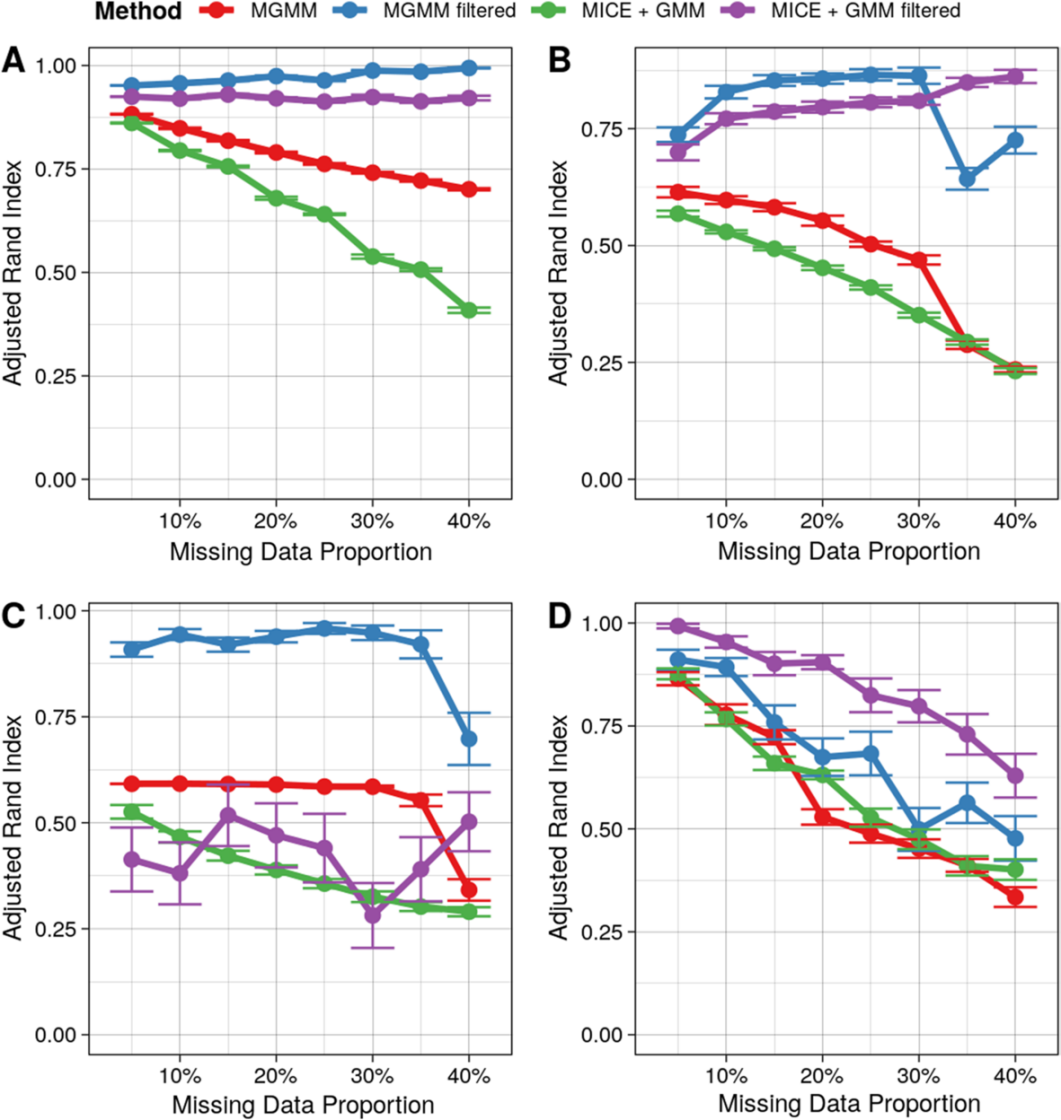
Performances of MICE-filtered and MGMM-filtered on four Benchmark Data Sets. The adjusted Rand index as a function of the missing data proportion for **A** the Four Component Mixture of Bivariate Gaussians simulation, **B** Cancer RNA-Seq Data Set, **C** Simulated Multi-trait GWAS Summary Statistics, **D** 2nd Example of Real Multi-trait GWAS Summary Statistics for Cardiovascular Risk Factors. Error bars represent the standard error of the mean across 40 simulation replicates

By effectively removing poorly classifiable observations from consideration, filtering is expected to improve the clustering quality, but only if those observations with high assignment uncertainty are correctly identified. Therefore, the comparative performance of MGMM-filtered and MICE-filtered provides an indication of how well each strategy was able to identify those observations with high cluster assignment uncertainty. We present the performances of the two methods on four data sets in Fig. 7.

#### Four component mixture of bivariate Gaussians

For the Gaussian mixture simulation data set (Fig. 7A), filtering out unassignable observations strikingly improved the classification accuracy of both MICE and MGMM. However, MGMM-filtered performed better for all missing data ratios. Thus, when the data are in fact generated by a GMM, MGMM correctly assesses cluster assignment uncertainty, providing users with a mechanism for identifying observations with low-confidence cluster assignments.

#### RNA sequence data from cancer patients

For the cancer RNA-Seq data set (Fig. 7B), entropy-based filtering again significantly improved the performance of both methods, suggesting that assignment entropy provides a reliable method for identifying unassignable observations. Note that the filtered data set contained sufficiently many observations to correctly evaluate performance (see Appendix 5). MGMM-filtered outperformed MICE-filtered at lower missingness, while MICE-filtered performed better when the missing data proportion exceeded 30%. The same trend was observed for the unfiltered versions of MGMM and MICE. This example demonstrates that even when the underlying distribution is not a GMM, MGMM is able to accurately assess cluster assignment uncertainty at practical missing ratios.

#### GWAS summary statistics

For the GWAS summary statistic data sets, the comparative performances of the two methods depend on the structure of the data. On the simulated multi-trait GWAS summary statistics (Fig. 7C), filtering drastically improved the performance of MGMM, whereas filtering did little, if anything, to improve the performance of MICE. This suggest that MICE-based imputation entropy was not an effective gauge of assignment uncertainty for these data. The non-linearity and absence of correlation among the variables probably explains the poor performance of MICE.

On the 2nd example of real GWAS summary statistics for cardiovascular risk factors, MICE-filtered performed best overall, and entropy-based filtering improved the performance of MICE more so than the performance of MGMM. The unfiltered versions of MICE and MGMM performed comparably. The strong correlations among the traits studied likely explains the good performance of MICE-filtered for these data. It is also important to note that, for the GWAS data sets, the reference labels used to compute the adjusted rand index are not the true classes *per se*, but rather the clustering obtained on complete data (see the "Methods" section). Therefore, the performance assessment in this example is more a measure of the robustness of the clustering procedure to the presence of missing data than a measure of the capacity to identify true underlying classes.

#### Filtered observations

Importantly, filtering out unassignable observations based on entropy did not strongly enrich the remaining data for complete cases (see Supplementary Fig. 5). Therefore, the general improvements in performance observed with filtering cannot be trivially explained by the selective removal of incomplete observations, and point instead to the accurate identification of observations that could plausibly have arisen from more than 1 cluster.

#### Running time benchmark

**Fig. 8:**
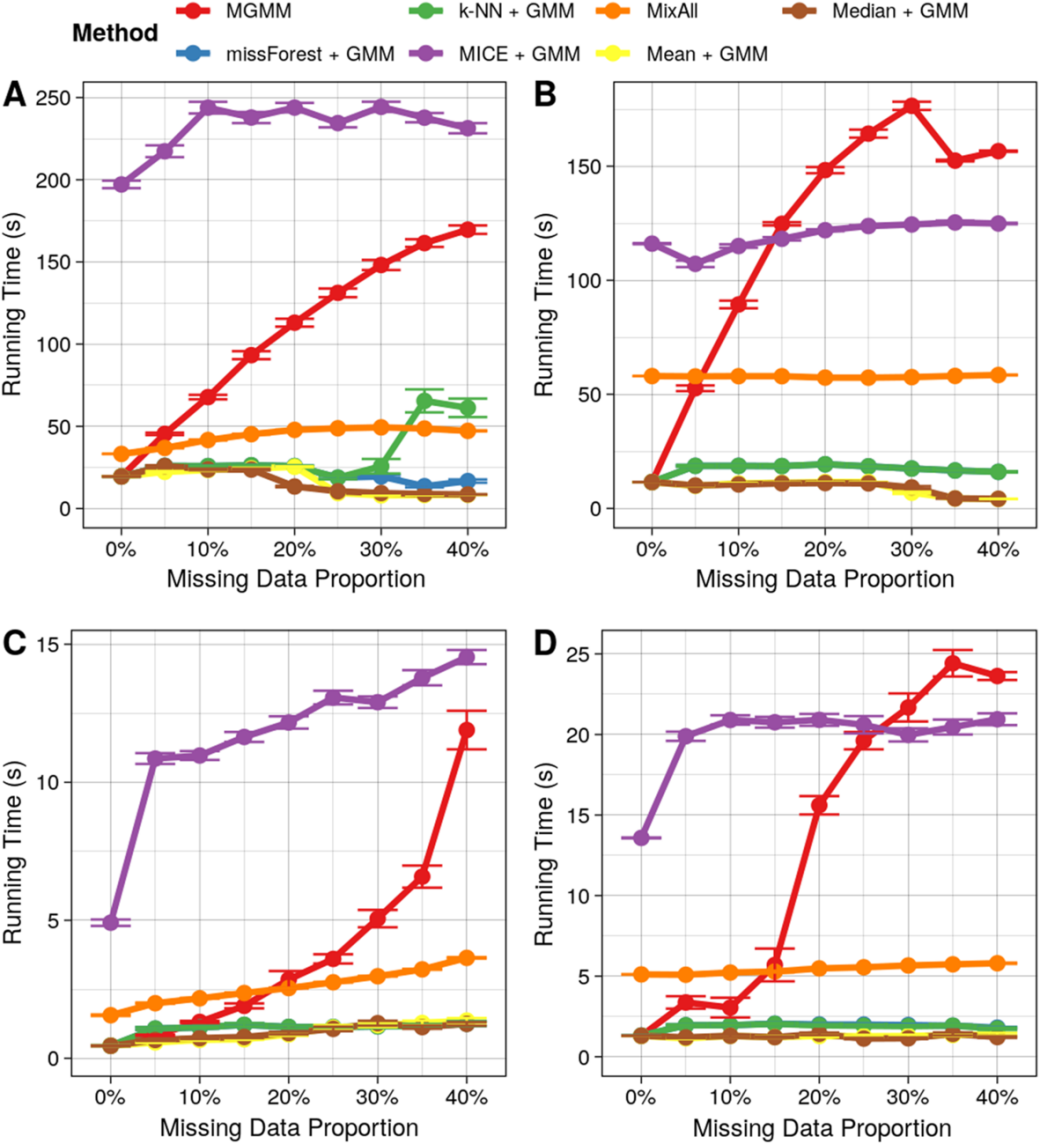
Comparison of computation time for studied methods. The computation time as a function of the missing data proportion for **A** the Four Component Mixture of Bivariate Gaussians simulation, **B** Cancer RNA-Seq Data Set, **C** Simulated Multi-trait GWAS Summary Statistics, **D** 2nd Example of Real Multi-trait GWAS Summary Statistics for Cardiovascular Risk Factors. Error bars represent the standard error of the mean across 40 simulation replicates

In terms of running time (see Fig. 8), simple and biased imputation scheme such as mean and median imputation were consistently fastest. The running time of k-NN and random forest remained low for all missing rate. Mixall was slower than MGMM on complete data and for realistic missing rate. MGMM was competitive for the missingness rates usually encountered in real data ($\overset{\sim }{1}0\%$) but its running time increased steeply as the proportion of missing data became large. MICE followed by MGMM was the slowest method overall and was slow even for low missingness rates. This naturally follows from needing to perform multiple rounds of imputation followed by GMM estimation with MICE. Although MICE and MGMM were generally the slowest methods, these are also the only methods that provide a mechanism for identifying and filtering out observations with high assignment uncertainty. Moreover, to put the computation cost into perspective, the longest observed running time was 344 seconds, which remains tractable (obtained with MICE on the Mixture of Bivariate Gaussians example, Fig. 8A).

## Discussion

We conducted a comparative benchmark to assess the capacity of MGMM versus MixAll and standard GMM after imputation to correctly identify true cluster assignments in data containing missing values. We established that for data sets following a distribution close to a GMM, MGMM is able to recover the true class assignment more accurately than imputation followed by standard GMM. When the underlying data generating process is in fact a GMM, then as a correctly specified maximum likelihood procedure, MGMM is optimal. MGMM consistently outperformed the other existing GMM implementation that allows for missing data (i.e. MixAll), except when the proportion of missing data became excessive. The better performance of MGMM at low levels of missingness is likely because MGMM places no restrictions on the form of the covariance matrix. At high levels of missingness, adopting the parsimonious assumption of a diagonal covariance structure, as is done by MixAll, can be advantageous. However, for a fixed proportion of missing data, MGMM should match or exceed the performance of MixAll as sample sizes increases. In addition, MGMM correctly assess its level of uncertainty in clustering assignments, providing a mechanism for identifying and separating out observations whose cluster assignments are unreliable.

GMMs are not well-suited to all clustering tasks. Direct application of MGMM was less effective than non-linear imputation, via kNN or random forests, followed by standard GMM in cases where the clusters present in the observed data were poorly differentiated, or the missingness was high (e.g. $40\%$). This observation emphasizes the need to assess the appropriateness of a GMM before applying MGMM to a clustering problem. Since kNN and random forest imputation, followed by standard GMM, were typically competitive with MGMM in the real data examples, these methods may be used to perform sensitivity analysis on the final cluster assignments. On the other hand, standard GMM following kNN or random forest imputation will not appropriately propagate uncertainty due to missing data. This can lead to inaccurate estimates of the posterior membership probabilities, particularly for observations with multiple missing elements, and failure to identify observations whose cluster assignments are unreliable. Thus, an approach such as MGMM-filtered, which accurately assesses assignment uncertainty and removes unclassifiable observations from consideration, may be more reliable. The framework proposed by [6], and elaborated upon here, of using an EM-type algorithm to fit mixture models in the presence of both missing data and unknown class assignments, may be extended to estimates mixtures of non-Gaussian distributions. Extending MGMM to estimate such mixtures in the presence of missing data is among our future directions.

## Conclusion

We have presented MGMM, a powerful, general purpose R package for maximum likelihood-based estimation of GMMs in the presence of missing data, and demonstrated that MGMM often outperforms both MixAll and imputation followed by standard GMM on various real and simulated data sets. In contrast to estimation after imputation, MGMM uses the ECM algorithm to efficiently and unbiasedly obtain the maximum likelihood estimates of all model parameters while properly accounting for the uncertainty introduced by the presence of missing values; and in contrast to MixAll, which also employs maximum likelihood estimation, MGMM does not assume the data are uncorrelated. To our knowledge, MGMM is the only publicly available method for fitting GMMs that properly accounts for missing data without imposing simplifying assumptions, and our benchmark is the first extensive study of how estimating GMMs while properly accounting for missing data compares with the *ad hoc* procedure of estimation after imputation. In addition, the supporting information (Additional file 1) provides a clear step-by-step derivation of our ECM algorithm, providing a foundation for extending this work to missingness-aware mixtures of other distributions. The functionalities of the MGMM package [7] are carefully documented and comprise: the generation of random data under a specified GMM, the fitting of GMMs to data sets containing missing values, the drawing of multiple imputations for a fitted model, and the computation of a panel of clustering criteria to identify the optimal number of clusters.

## Supplementary information

**Additional file 1:** Detailed derivation; discussion of assumptions, cluster-number selection, and multiple-imputation; additional simulation materials.

**Additional file 2.** Replication scripts and benchmark data.

## Data Availability

MGMM is available as an R package on CRAN: https://CRAN.R-project.org/package=MGMM. The data used in this study are either publicly available or were randomly generated according to the procedure detailed in the Materials and Methods. We provide an archive (Additional file 2) containing replication scripts and data.
