# Correction: Fitting Gaussian mixture models on incomplete data

**DOI:** 10.1186/s12859-022-04808-6

**Published:** 2022-06-27

**Authors:** Zachary R. McCaw, Hugues Aschard, Hanna Julienne

**Affiliations:** 1 School of Public Health, Harvard T.H. Chan, 677 Huntington Ave, Boston, MA 02115 USA; 2 Department of Computational Biology, Institut Pasteur, Université de Paris, 25-28 Rue du Dr Roux, 75015 Paris, France

## Correction: BMC Bioinformatics (2022) 23:208 https://doi.org/10.1186/s12859-022-04740-9

Following the publication of the original article [1], the authors would like to add additional information to the funding note. The updated funding note is given below.

Funding:

This research was supported by the Agence Nationale pour la Recherche (ANR-20-CE36-0009-02). This work is supported in part by funds from the National Science Foundation (NSF: # 1636933 and # 1920920).

The original article [1] has been corrected.
