# Supplementary material for: Fitting Gaussian mixture models on incomplete data
Source: BMC Bioinformatics. 2022 Jun 1;23:208. doi: 10.1186/s12859-022-04740-9 (PMC9158227; doi:10.1186/s12859-022-04740-9)
Supplement: Supplementary file 1 — Additional file 1: Detailed derivation; discussion of assumptions, cluster-number selection, and multiple-imputation; additional simulation materials. [file 12859_2022_4740_MOESM1_ESM.pdf]

Supporting Information for Fitting Gaussian Mixture Models on Incomplete Data

Zachary R. McCaw, Hugues Aschard, Hanna Julienne

May 30, 2022

1 Complete derivation

Here we present a complete derivation for estimation of Gaussian mixture models (GMMs) in the presence of missing data. We start by defining the objective function optimized by the expectation conditional maximization (ECM) algorithm, which is expressed in terms of three key quantities: the *responsibilities*, the *working response*, and the *working residual outer product*. Next, we describe the optimization routine step-by-step. Finally, we discuss the assumptions required of the missing data in order for the estimation procedure to be unbiased.

1.1 Objective function

The log likelihood of the observed data is:

$$\ell(\boldsymbol{\pi}, \boldsymbol{\theta}) \propto \sum_{i=1}^{n} \ln \left\{ \sum_{j=1}^{k} f(\boldsymbol{y}_i^{\text{obs}} | \boldsymbol{\mu}_j, \boldsymbol{\Sigma}_j) \pi_j \right\}, \tag{1}$$

where $\boldsymbol{y}_i^{\text{obs}}$ represents only the observed components of the complete-data vector $\boldsymbol{y}_i$. However, due to the presence of a sum within the logarithm, there are no closed-form solutions for optimizing (1) in general. Here we develop the objective function optimized by the ECM algorithm, which is a lower bound on the observed data log likelihood [3].

Define the $d \times d$ *residual outer product* matrix as:

$$\boldsymbol{V}_{ij} = z_{ij}(\boldsymbol{y}_i - \boldsymbol{\mu}_j)(\boldsymbol{y}_i - \boldsymbol{\mu}_j)'.$$

Let $\boldsymbol{\theta} = (\boldsymbol{\mu}_1, \boldsymbol{\Sigma}_1, \cdots, \boldsymbol{\mu}_k, \boldsymbol{\Sigma}_k)$ collect the means and covariances of the component normal distributions. *Complete data* refers to the case where all elements of $\boldsymbol{y}_i$ and all cluster assignments $\boldsymbol{z}_i$ are observed. The complete data log likelihood is:

$$\ell(\boldsymbol{\pi}, \boldsymbol{\theta}) \propto \sum_{i=1}^{n} \sum_{j=1}^{k} z_{ij} \ln \pi_j - \frac{1}{2} \sum_{i=1}^{n} \sum_{j=1}^{k} z_{ij} \ln \det(\boldsymbol{\Sigma}_j) - \frac{1}{2} \sum_{i=1}^{n} \sum_{j=1}^{k} \text{tr}\big(\boldsymbol{\Sigma}_j^{-1} \boldsymbol{V}_{ij}\big). \tag{2}$$

Let $\boldsymbol{\pi}^{(r)}$ and $\boldsymbol{\theta}^{(r)}$ denote the current estimates of $\boldsymbol{\pi}$ and $\boldsymbol{\theta}$, and let $\mathcal{D}_{\text{obs}} = \cup_{i=1}^{n}\{\boldsymbol{y}_i^{\text{obs}}\}$ denote the observed data. The *ECM objective* is defined as the expectation of the complete data log likelihood (2) given the observed data and the current parameter states:

$$Q(\boldsymbol{\pi}, \boldsymbol{\theta}|\boldsymbol{\pi}^{(r)}, \boldsymbol{\theta}^{(r)}) \equiv \mathbb{E}\big\{\ell(\boldsymbol{\pi}, \boldsymbol{\theta})\big|\mathcal{D}_{\text{obs}}; \boldsymbol{\pi}^{(r)}, \boldsymbol{\theta}^{(r)}\big\}.$$

Define the *responsibility* of the jth cluster for the ith observation as the current conditional probability of membership given the observed data:

$$\hat{\gamma}_{ij}^{(r)} \equiv \mathbb{P}(z_{ij} = 1|\boldsymbol{y}_i^{\text{obs}}, \boldsymbol{\pi}^{(r)}, \boldsymbol{\theta}^{(r)}). \tag{3}$$

From Bayes' theorem, the responsibility is expressible as:

$$\hat{\gamma}_{ij}^{(r)} = \frac{\mathbb{P}(\boldsymbol{y}_i^{\text{obs}}|z_{ij} = 1, \boldsymbol{\pi}^{(r)}, \boldsymbol{\theta}^{(r)})\mathbb{P}(z_{ij} = 1|\boldsymbol{\pi}^{(r)})}{\mathbb{P}(\boldsymbol{y}_i^{\text{obs}}|\boldsymbol{\pi}^{(r)}, \boldsymbol{\theta}^{(r)})} = \frac{f(\boldsymbol{y}_i^{\text{obs}}|\boldsymbol{\mu}_j^{(r)}, \boldsymbol{\Sigma}_j^{(r)})\pi_j^{(r)}}{\sum_{l=1}^{k} f(\boldsymbol{y}_i^{\text{obs}}|\boldsymbol{\mu}_l^{(r)}, \boldsymbol{\Sigma}_l^{(r)})\pi_l^{(r)}}.$$

Here $f(\boldsymbol{y}_i^{\text{obs}}|\boldsymbol{\mu}_j^{(r)}, \boldsymbol{\Sigma}_j^{(r)})$ refers to the density of the observed elements $\boldsymbol{y}_i^{\text{obs}}$ of $\boldsymbol{y}_i$ given the current mean $\boldsymbol{\mu}_j^{(r)}$ and covariance $\boldsymbol{\Sigma}_j^{(r)}$ of the jth cluster.

Given membership to the jth cluster, the joint distribution of the observed $\boldsymbol{y}_i^{\text{obs}}$ and missing $\boldsymbol{y}_i^{\text{miss}}$ elements of $\boldsymbol{y}_i$ is:

$$\begin{pmatrix} \boldsymbol{y}_i^{\text{obs}} \\ \boldsymbol{y}_i^{\text{miss}} \end{pmatrix}\Bigg|(z_{ij} = 1) \sim N\left\{\begin{pmatrix} \boldsymbol{\mu}_{\text{obs},j} \\ \boldsymbol{\mu}_{\text{miss},j} \end{pmatrix}, \begin{pmatrix} \boldsymbol{\Sigma}_{\text{obs},j} & \boldsymbol{\Sigma}_{\text{obs,miss},j} \\ \boldsymbol{\Sigma}_{\text{miss,obs},j} & \boldsymbol{\Sigma}_{\text{miss},j} \end{pmatrix}\right\}.$$

For the ith observation, define the jth *working response vector* as:

$$\hat{\boldsymbol{y}}_{ij}^{(r)} \equiv \mathbb{E}\left\{\begin{pmatrix} \boldsymbol{y}_i^{\text{obs}} \\ \boldsymbol{y}_i^{\text{miss}} \end{pmatrix}\Bigg| z_{ij} = 1, \boldsymbol{y}_i^{\text{obs}}; \boldsymbol{\pi}^{(r)}, \boldsymbol{\theta}^{(r)}\right\} = \begin{pmatrix} \boldsymbol{y}_i^{\text{obs}} \\ \hat{\boldsymbol{y}}_{ij}^{\text{miss},(r)} \end{pmatrix}, \tag{4}$$

where $\hat{\boldsymbol{y}}_{ij}^{\text{miss},(r)}$ is the current conditional expectation of the missing elements of $\boldsymbol{y}_i$ under the assumption that observation i originated from cluster j:

$$\hat{\boldsymbol{y}}_{ij}^{\text{miss},(r)} = \mathbb{E}\big(\boldsymbol{y}_i^{\text{miss}}|z_{ij} = 1, \boldsymbol{y}_i^{\text{obs}}; \boldsymbol{\pi}^{(r)}, \boldsymbol{\theta}^{(r)}\big) = \boldsymbol{\mu}_{\text{miss},j}^{(r)} + \big\{\boldsymbol{\Sigma}_{\text{miss,obs},j}\boldsymbol{\Sigma}_{\text{obs},j}^{-1}\big\}^{(r)}(\boldsymbol{y}_i^{\text{obs}} - \boldsymbol{\mu}_{\text{obs},j}^{(r)}).$$

Finally, define the *working residual outer product* as:

$$\begin{aligned} \hat{\boldsymbol{V}}_{ij}^{(r)}(\boldsymbol{\mu}_j) &\equiv \mathbb{E}\big(\boldsymbol{V}_{ij}|\boldsymbol{y}_i^{\text{obs}}; \boldsymbol{\pi}^{(r)}, \boldsymbol{\theta}^{(r)}\big) \\ &= \hat{\gamma}_{ij}^{(r)}\left\{\big(\hat{\boldsymbol{y}}_{ij}^{(r)} - \boldsymbol{\mu}_j\big)\big(\hat{\boldsymbol{y}}_{ij}^{(r)} - \boldsymbol{\mu}_j\big)' + \begin{pmatrix} 0 & 0 \\ 0 & \boldsymbol{\Lambda}_{j,TT}^{(r),-1} \end{pmatrix}\right\}. \end{aligned} \tag{5}$$

The working residual outer product is the current conditional expectation of the residual outer product given the observed data.

In terms of the responsibility and the working residual outer product, the ECM objective is:

$$Q(\boldsymbol{\pi}, \boldsymbol{\theta}|\boldsymbol{\pi}^{(r)}, \boldsymbol{\theta}^{(r)}) \equiv \sum_{i=1}^{n}\sum_{j=1}^{k}\hat{\gamma}_{ij}^{(r)}\ln\pi_j - \frac{1}{2}\sum_{i=1}^{n}\sum_{j=1}^{k}\hat{\gamma}_{ij}^{(r)}\ln\det(\boldsymbol{\Sigma}_j) - \frac{1}{2}\sum_{i=1}^{n}\sum_{j=1}^{k}\text{tr}\big(\boldsymbol{\Sigma}_j^{-1}\hat{\boldsymbol{V}}_{ij}^{(r)}\big). \tag{6}$$

In contrast to the complete data log likelihood in (2), the ECM objective (6) is a function of the observed data only.

1.2 Optimization

ECM differs from classic EM in that the M-step is partitioned into a sequence of conditional maximizations [5]. In the present case, estimation of the means ($\boldsymbol{\mu}_j$), followed by the covariances ($\boldsymbol{\Sigma}_j$), and finally the cluster membership probabilities $\boldsymbol{\pi}$. The advantage of ECM is that each conditional maximization is available in closed form.

The optimization procedure is outlined in Algorithm 1. Initial estimates of $\boldsymbol{\pi}$ and $\boldsymbol{\theta}$ are required. The approach adopted by `MGMM` is to perform an initial k-means clustering on complete observations [1]. $\hat{\boldsymbol{\pi}}$ is estimated as the proportion of the complete observations assigned to each cluster, and $\boldsymbol{\theta}$ is estimated by calculating the within-cluster means and covariances.

Update equations were derived by differentiating the ECM objective in (6) and solving the resulting score equations. The update for $\boldsymbol{\mu}_j$ is:

$$\boldsymbol{\mu}_j^{(r+1)} \leftarrow \frac{1}{n_j^{(r)}} \sum_{i=1}^{n} \hat{\gamma}_{ij}^{(r)} \hat{\boldsymbol{y}}_{ij}^{(r)}, \tag{7}$$

where $\hat{\gamma}_{ij}^{(r)}$ is the current responsibility (3) of the jth cluster for the ith observation,

$$n_j^{(r)} = \sum_{i=1}^{n} \hat{\gamma}_{ij}^{(r)}$$

is the total responsibility of the jth cluster, and $\hat{\boldsymbol{y}}_{ij}^{(r)}$ is the current working response vector (4). Note that (7) takes the form of a responsibility-weighted average of the working response vectors. Similarly, the update for $\boldsymbol{\Sigma}_j$ is:

$$\boldsymbol{\Sigma}_j^{(r+1)} \leftarrow \frac{1}{n_j^{(r)}} \sum_{i=1}^{n} \hat{\boldsymbol{V}}_{ij}^{(r)} (\boldsymbol{\mu}_j^{(r+1)}),$$

which is a responsibility-weighted average of the working residual outer products (5).

Given the updated means $\{\boldsymbol{\mu}_j^{(r+1)}\}$ and covariates $\{\boldsymbol{\Sigma}_j^{(r+1)}\}$, the new responsibilities are:

$$\hat{\gamma}_{ij}^{(r+1)} \leftarrow \frac{f(\boldsymbol{y}_i^{\text{obs}} | \boldsymbol{\mu}_j^{(r+1)}, \boldsymbol{\Sigma}_j^{(r+1)}) \pi_j^{(r)}}{\sum_{l=1}^{k} f(\boldsymbol{y}_i^{\text{obs}} | \boldsymbol{\mu}_l^{(r+1)}, \boldsymbol{\Sigma}_l^{(r+1)}) \pi_l^{(r)}}. \tag{8}$$

Finally, the update for π_j is:

$$\hat{\pi}_j^{(r+1)} \leftarrow \frac{1}{n} \sum_{i=1}^{n} \hat{\gamma}_{ij}^{(r+1)} = \frac{n_j^{(r+1)}}{n}.$$

After all parameters have been updated, the current value of the ECM objective $Q^{(r+1)} = Q(\boldsymbol{\pi}^{(r+1)}, \boldsymbol{\theta}^{(r+1)} | \boldsymbol{\pi}^{(r+1)}, \boldsymbol{\theta}^{(r+1)})$ is calculated from (6), and the algorithm continues until the increment $Q^{(r+1)} - Q^{(r)}$ in the objective falls below a pre-specified tolerance ϵ.

Algorithm 1 ECM for GMMs

Initialize $\boldsymbol{\pi}^{(0)}$ and $\boldsymbol{\theta}^{(0)}$.
repeat
    Update the cluster responsibilities to $\hat{\gamma}_{ij}^{(r)}$ using (8).
    Construct the working vectors $\hat{\boldsymbol{y}}_{ij}^{(r)}$ using (4).
    Update the cluster means $\boldsymbol{\mu}_j^{(r+1)} \leftarrow \{n_j^{(r)}\}^{-1} \sum_{i=1}^{n} \hat{\gamma}_{ij}^{(r)} \hat{\boldsymbol{y}}_{ij}^{(r)}$.
    Update the cluster covariances $\boldsymbol{\Sigma}_j^{(r+1)} \leftarrow \{n_j^{(r)}\}^{-1} \sum_{i=1}^{n} \hat{\boldsymbol{V}}_{ij}^{(r)}(\boldsymbol{\mu}_j^{(r+1)})$.
    Update the cluster membership probabilities $\hat{\pi}_j^{(r+1)} \leftarrow n_j^{(r)}/n$.
    Calculate the final objective $Q^{(r+1)} = Q(\boldsymbol{\pi}^{(r+1)}, \boldsymbol{\theta}^{(r+1)} | \boldsymbol{\pi}^{(r+1)}, \boldsymbol{\theta}^{(r+1)})$.
until $Q^{(r+1)} - Q^{(r)} < \epsilon$.

1.3 Assumption on missingness

Standard approaches to addressing missing data, including complete case analysis and naive mean or median imputation, tacitly assume that the data are missing completely at random (MCAR). Under MCAR, whether an element of data is missing is completely independent of its value. However, unbiased estimation via the maximum likelihood-based approach presented here only requires that the missingness occurs at random (MAR), which is a weaker and more plausible assumption. Under MAR, whether an element of data is missing is independent of its value conditional on those elements of the data that are observed [4].

To elaborate on this assumption, let $R_{il} = 1$ if the lth component of the ith observation (that is, Y_{il}) is observed, and define the response indicator vector $\boldsymbol{r}_i = \text{vec}(R_{i1}, \cdots, R_{ip})$. For the ith subject, let $\mathcal{Y}_i^{\text{obs}}$ denote the indices of the elements of $\boldsymbol{y}_i$ that are observed, and $\mathcal{Y}_i^{\text{miss}}$ the indices of the elements that are missing. Partition $\boldsymbol{y}_i$ as $(\boldsymbol{y}_i^{\text{obs}}, \boldsymbol{y}_i^{\text{miss}})$, where $\boldsymbol{y}_i^{\text{obs}}$ contains the observed elements of $\boldsymbol{y}_i$, and $\boldsymbol{y}_i^{\text{miss}}$ contains the missing elements. The MAR assumption requires that, for each observation i and all missing elements $l \in \mathcal{Y}_i^{\text{obs}}$, the response indicator R_{il} is conditionally independent of Y_{il} given that observation's observed data $\boldsymbol{y}_i^{\text{obs}}$. For example, MAR holds under the generalized linear model $g\{\mathbb{P}(R_{il} = 1 | \boldsymbol{y}_i^{\text{obs}})\} = \boldsymbol{\beta}' \boldsymbol{y}_i^{\text{obs}}$, for some regression coefficient $\boldsymbol{\beta}$. MAR fails if the missing occurs not at random (MNAR), for example if $R_{il} = \mathbb{I}(|Y_{il}| \leq \tau)$ for some threshold τ, or if $g\{\mathbb{P}(R_{il} = 1 | \boldsymbol{y}_i^{\text{obs}}, Y_{il})\} = \boldsymbol{\beta}' \boldsymbol{y}_i^{\text{obs}} + \alpha Y_{il}$ for $\alpha \neq 0$. That is, the data are MNAR if $R_{il} \not\perp Y_{il} | \boldsymbol{y}_i^{\text{obs}}$. Unbiased estimation under MNAR requires knowledge of the missing data mechanism, which is typically not available.

2 Selecting the number of clusters

In some settings, such as the RNA-seq data set, the number of clusters is suggested by the number of classes present (i.e. tumor types). In others, such as clustering GWAS summary statistics, the number of clusters is unknown. The `ChooseK` function from the `MGMM` package aims to assist in choosing the number of clusters k. For a range of possible k, bootstrap samples of the input data are selected, a GMM is fit, and several cluster quality metrics are calculated. These include the Bayesian Information Criterion, the Calinski-Harabaz Index, the Davies-Bouldin Index, and the silhouette width. For each candidate k, the mean and standard error, across bootstrap data sets, of the cluster quality metrics are recorded. For each metric, the cluster number leading to the optimal quality k_{opt} and the smallest cluster number whose quality was within 1 standard error of optimal k_{1se} are determined. Which clustering criterion to optimize may depend on the application. Absent other considerations, we suggest optimizing the silhouette width, which typically does not select an excessive number of clusters, and using k_{1se} in the interest of parsimony. For additional discussion on choosing the number of clusters, see [2].

3 Multiple imputation-based inference

Suppose the parameters $(\boldsymbol{\pi}, \boldsymbol{\theta})$ of a GMM have been estimated from the observed data, however interest ultimately lies in estimating $\boldsymbol{\vartheta}$, whose estimator $\hat{\boldsymbol{\vartheta}}(\mathcal{D})$ is a function of the complete data $\mathcal{D}$. For example, $\boldsymbol{\vartheta}$ might be the marginal mean of the outcome vectors, as estimated by: $\hat{\boldsymbol{\vartheta}}(\mathcal{D}) = \frac{1}{n}\sum_{i=1}^{n}\boldsymbol{y}_i$. Let $\hat{v}(\mathcal{D}) = \hat{\mathbb{V}}\{\hat{\boldsymbol{\vartheta}}(\mathcal{D})\}$ denote the estimated sampling variance (i.e. the square of the standard error) of $\hat{\boldsymbol{\vartheta}}(\mathcal{D})$. To perform multiple imputation-based inference on $\boldsymbol{\vartheta}$, M stochastic imputations $\{\mathcal{D}^{(1)}, \cdots, \mathcal{D}^{(M)}\}$ of the data are drawn. For the mth imputation, let $\hat{\boldsymbol{\vartheta}}^{(m)} = \hat{\boldsymbol{\vartheta}}(\mathcal{D}^{(m)})$ denote the parameter estimate and $\hat{v}^{(m)} = \hat{v}(\mathcal{D}^{(m)})$ its sampling variance. The final estimate of $\boldsymbol{\vartheta}$ is simply the average estimate across imputations:

$$\hat{\boldsymbol{\vartheta}} = \frac{1}{M}\sum_{m=1}^{M}\hat{\boldsymbol{\vartheta}}^{(m)}$$

A valid estimate for the variance of $\hat{\boldsymbol{\vartheta}}$ is obtained using Rubin's rules:

$$\hat{\mathbb{V}}(\hat{\boldsymbol{\vartheta}}) = \frac{1}{M}\sum_{m=1}^{M}\hat{v}^{(m)} + \frac{1}{M-1}\sum_{m=1}^{M}\big(\hat{\boldsymbol{\vartheta}}^{(m)} - \hat{\boldsymbol{\vartheta}}\big)^{\otimes 2}.$$

Notice that the estimated sampling variance of $\hat{\boldsymbol{\vartheta}}$ is the sum of two terms. The first term is a measure of within-imputation variability, whereas the second term is a measure of between-imputation variability. Using $\hat{\boldsymbol{\vartheta}}$ and $\hat{\mathbb{V}}(\hat{\boldsymbol{\vartheta}})$, standard inference procedures may now be applied. For example, an asymptotically valid Wald statistic for assessing $H_0 : \boldsymbol{\vartheta} = \boldsymbol{\vartheta}_0$ is:

$$T_W = \big(\hat{\boldsymbol{\vartheta}} - \boldsymbol{\vartheta}_0\big)'\big\{\hat{\mathbb{V}}(\hat{\boldsymbol{\vartheta}})\big\}^{-1}\big(\hat{\boldsymbol{\vartheta}} - \boldsymbol{\vartheta}_0\big).$$

Under the null hypothesis, T_W follows an asymptotic $\chi_d^2(0)$ distribution with $d = \dim(\boldsymbol{\vartheta})$ degrees of freedom.

4 Additional analyses of GWAS summary statistics

Here we present two additional examples of clustering summary statistics from real GWAS of cardiovascular disease risk factors (Supplementary Figures 1 and 2). In both cases, the underlying data generating mechanism is unknown, but is unlikely to be a GMM. One cluster (green), corresponding to SNPs associated with BMI, is well differentiated, while the remaining two clusters (blue and gold) are poorly resolved, and it is not obvious in either case that there truly are thee clusters present in the observed data. In this context, non-linear imputation, via kNN or random forests, followed by standard GMM outperformed MGMM. This is not surprising: when the observed data arise from a distribution that is far from a GMM, using MGMM to circumvent imputation is unlikely to succeed. These examples underscore the point made in the conclusions that GMMs, and MGMM in particular, are not suited to all clustering tasks.

5 Comparison of the MICE-filtered and MGMM-filtered procedures

We compared the fraction of observations deemed unassignable by the MICE-filtered and MGMM-filtered to ensure that filtering did not lead to an over-enrichment of complete observations, as this might have artificially increased the apparent performance. Supplementary Figure 3 displays the entropy distribution for the MICE and MGMM method and the fraction of filtered data point by entropy treshold. Supplementary Figure 4 presents the fraction of observations deemed unassignable from the cancer RNA-Seq data set, and the second 5-trait real GWAS benchmark. Note that, by design of the filtering procedure (see main material and methods), both methods remove the same proportion of observations, with minor variations due to the imprecision of the empirical distribution function for assignment entropy. As the missing data ratio increased, so too did the proportion of unassignable observations, likely due to a loss of information along coordinates important for differentiating the clusters.

Supplementary Figure 5 verifies that the proportion of complete observations remaining after filtering was not dramatically increased by either MGMM or MICE. The black dashed line on each panel represents the expected fraction of complete observations for a given missingness, assuming the missingness occurs completely at random. For missingness m and data of dimension d, this fraction is $f_{\text{complete}} = (1 - m)^d$.

6 Omnibus association test

The *omnibus test* is a classic, multi-trait test for assessing the association between a given SNP and any of a set of traits. Let $\mathbf{z}$ denote a d dimensional vector of

Z-scores quantifying the association between the SNP and the d traits. These are (asymptotically) normally distributed, with mean $\boldsymbol{\mu}$ and covariance $\boldsymbol{\Sigma}$:

$$\boldsymbol{z} \sim N\big(\boldsymbol{\mu}, \boldsymbol{\Sigma}\big).$$

Note that covariance arises among the Z scores when the traits are correlated. The null hypothesis of the omnibus test is that the means of all summary statistics are zero:

$$H_0 : \mu_1 = \mu_2 = \cdots = \mu_d = 0.$$

The alternative is that at least one mean is non-zero. The omnibus test statistic is the quadratic form:

$$T_{\text{omni}} \equiv \boldsymbol{z}'\boldsymbol{\Sigma}^{-1}\boldsymbol{z}.$$

Under the null, T_{omni} follows a central $\chi_d^2(0)$ distribution with d degrees of freedom:

$$T_{\text{omni}} \sim \chi_d^2(0). \tag{9}$$

7 Supplementary Figures

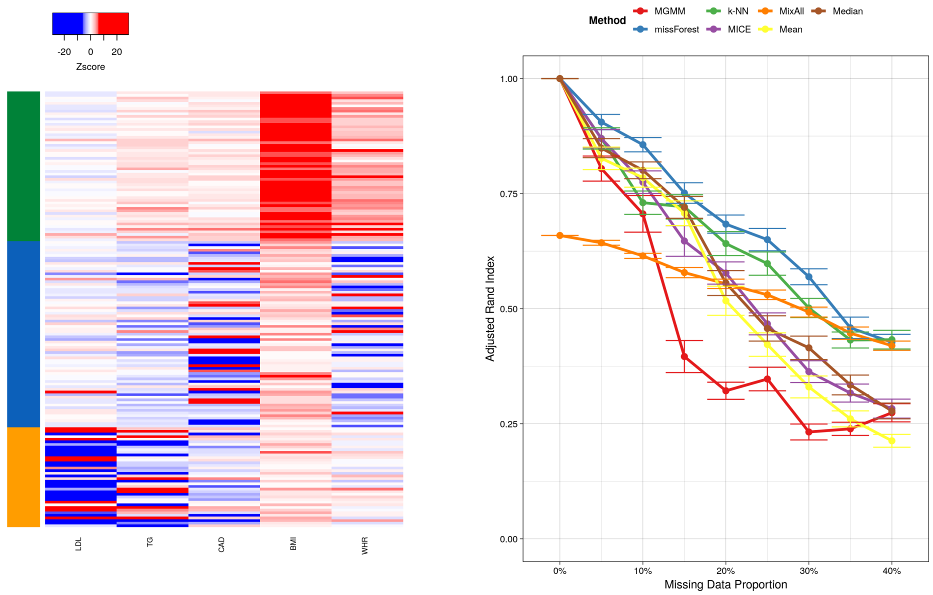

Supplementary Figure 1: **Benchmarking Real Multi-trait GWAS Summary Statistics for 5 Cardiovascular Risk Factors**. These were: body mass index (BMI), coronary artery disease (CAD), low density lipoprotein (LDL), triglycerides (TG), and waist-to-hip ratio (WHR). The left panel presents a heat map colored according to the standardized genetic effect, with SNPs as rows and traits as columns. The colorbar on the left represents the true cluster assignments. The right panel presents the adjusted Rand index as a function of the missing data proportion; a higher value indicates better agreement between the predicted and true cluster assignments, adjusting for chance. Error bars represent the standard error of the mean across 20 simulation replicates.

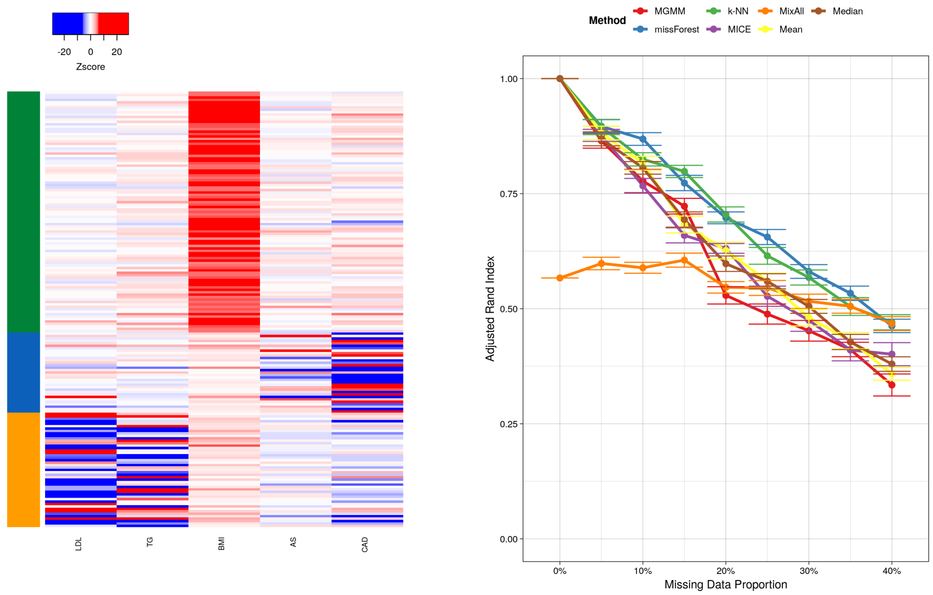

Supplementary Figure 2: **2nd Example of Benchmarking Real Multi-trait GWAS Summary Statistics for 5 Cardiovascular Risk Factors**. These were: any strokes (AS), body mass index (BMI), coronary artery disease (CAD), low density lipoprotein (LDL), triglycerides (TG). The left panel presents a heat map colored according to the normalized genetic effect, with SNPs as rows and traits as columns. The colorbar on the left represents the true cluster assignments. The right panel presents the adjusted Rand index as a function of the missing data proportion; a higher value indicates better agreement between the predicted and true cluster assignments, adjusting for chance. Error bars represent the standard error of the mean across 20 simulation replicates.

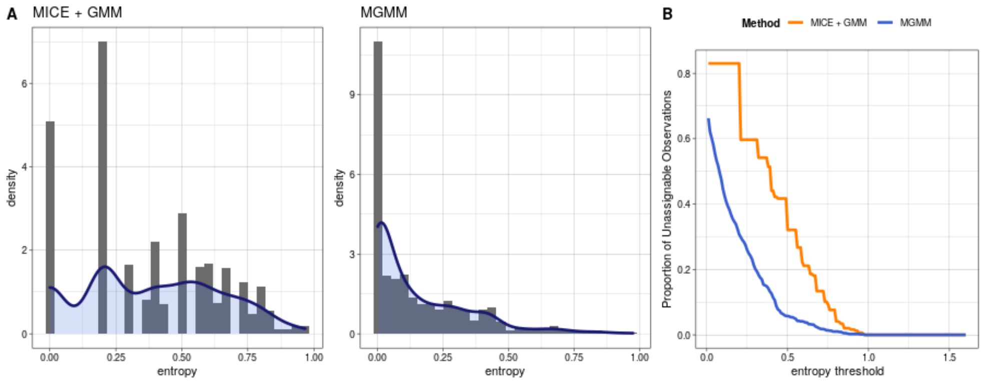

Supplementary Figure 3: **Comparison of Entropy Distributions of MICE and MGMM** A) Entropy distribution for each method on the Cancer RNA-Seq Data Set at a missing value ratio of 20%. B) Fraction of the data set deemed unassignable as a function of the entropy threshold.

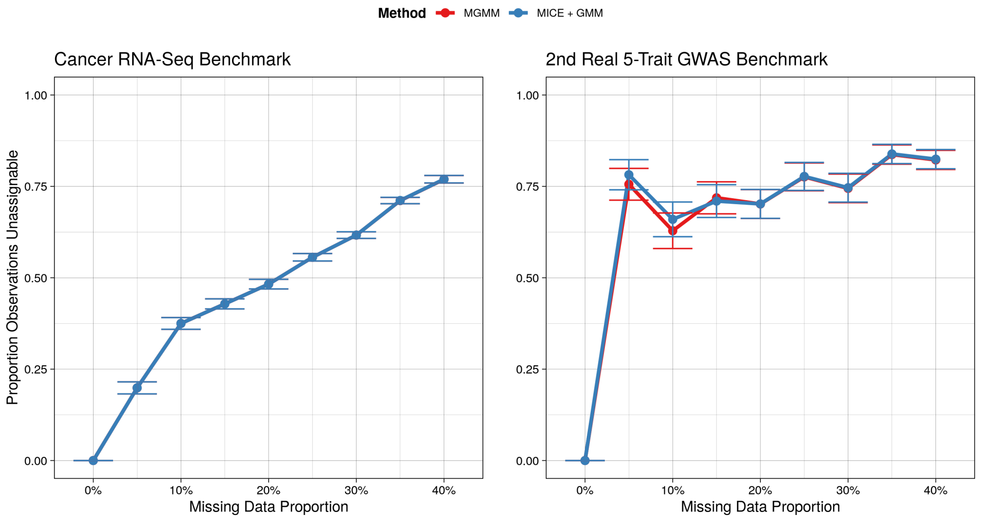

Supplementary Figure 4: **Proportion of Observations Deemed Unassignable as a Function of the Proportion of Missing Data.** By design of the filtering procedure, both MGMM-filtered and MICE-filtered should remove nearly the same proportion of observations.

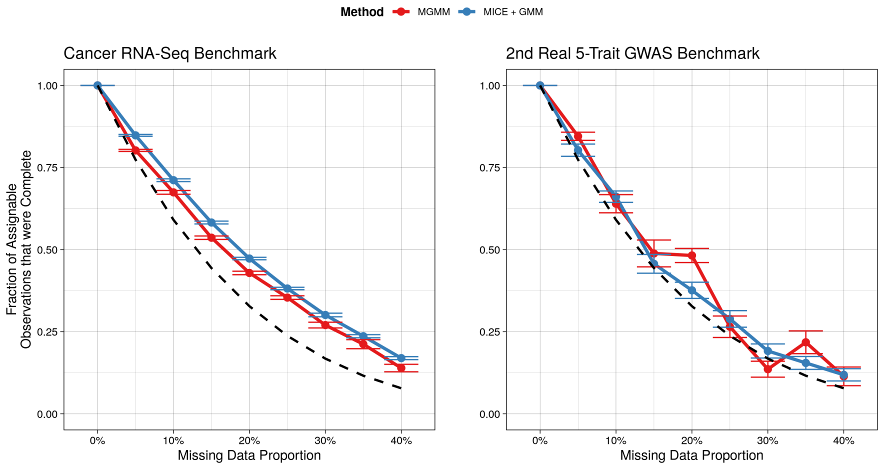

Supplementary Figure 5: Proportion of Complete Observations Remaining among the Assignable Observations. The expected fraction of complete observation is represented by the dashed black curve.

References

[1] CM Bishop. *Pattern Recognition and Machine Learning.* Information Science and Statistics. Springer Science+Business Media, 2006.

[2] T Hastie, R Tibshirani, and J Friedman. *The Elements of Statistical Learning: Data Mining, Inference, and Prediction.* Springer Series in Statistics. Springer Science+Business Media, 2nd edition, 2013.

[3] DR Hunter and K Lange. A tutorial on mm algorithms. *The American Statistician*, 58(1):30–37, 2004.

[4] RJA Little and DB Rubin. *Statistical Analysis with Missing Data.* John Wiley & Sons, 2nd edition, 2002.

[5] Xiao-Li Meng and Donald B Rubin. Maximum likelihood estimation via the ecm algorithm: A general framework. *Biometrika*, 80(2):267–278, 1993.
